# Correction: Unacylated Ghrelin Suppresses Ghrelin-Induced Neuronal Activity in the Hypothalamus and Brainstem of Male Rats

**DOI:** 10.1371/journal.pone.0102636

**Published:** 2014-07-07

**Authors:** The PLOS ONE Staff

## Notice of Republication

This article was republished on June 17, 2014, due to an error in the title. The publisher apologizes for this error. Please download this article again to view the corrected title and citation. The originally published, uncorrected article and the republished, corrected article are provided here for reference.

## Supporting Information

File S1 Originally published, uncorrected article
(PDF)

File S2 Republished, corrected article
(PDF)
