# Supplementary figures and images for: Unacylated Ghrelin Suppresses Ghrelin-Induced Neuronal Activity in the Hypothalamus and Brainstem of Male Rats
Source: PLoS One. 2014 May 22;9(5):e98180. doi: 10.1371/journal.pone.0098180 (PMC4031147; doi:10.1371/journal.pone.0098180)

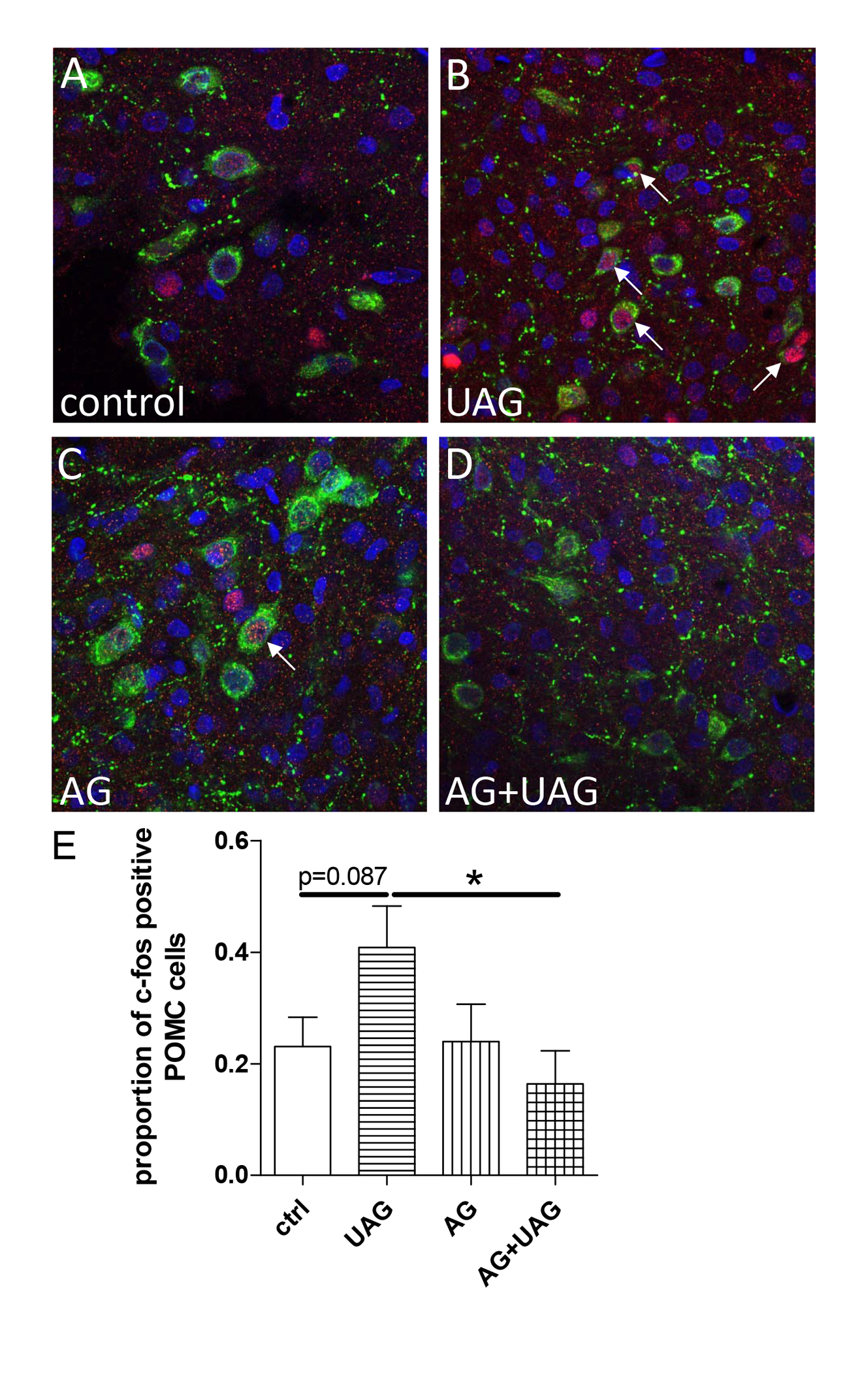

Supplement: Figure S1 — A. Co-localization of POMC/c-Fos after saline (A), AG (B), UAG (C) and AG+UAG (D) acute central treatment. POMC (green) and c-Fos (red) immunoreactivity was identified in sections of the hypothalamus using multi-label immunofluorescence immunohistochemistry. Nuclear staining (DAPI) is blue. Composite confocal laser-scanning microscope images are shown from a representative sections. The scale bar represents 20 µm. E. POMC/c-Fos co-localization ratio after saline, AG, UAG and combine treatment. Data are presented and mean ± SEM, * p<0.05 vs. saline. (TIF) [file pone.0098180.s001.tif]
