# Supplementary material for: Unacylated Ghrelin Suppresses Ghrelin-Induced Neuronal Activity in the Hypothalamus and Brainstem of Male Rats
Source: PLoS One. 2014 May 22;9(5):e98180. doi: 10.1371/journal.pone.0098180 (PMC4031147; doi:10.1371/journal.pone.0098180)
Supplement: Table S1 — Cycle threshold (Ct) values for the mRNA species amplified. (DOCX) [file pone.0098180.s002.docx]

Table S1: Cycle threshold (Ct) values for the mRNA species amplified

| **Gene** | **Ct ± SEM** |
| --- | --- |
| Hprt | 23.6 ± 0.13 |
| Agrp | 28.8 ± 0.19 |
| Pomc | 25.6 ± 0.15 |
| Mc3r | 27.6 ± 0.1 |
| Mc4r | 28.8 ± 0.16 |
| Bmp8b | 32.1 ± 0.22 |
| Ucp2 | 25.2 ± 0.21 |
