# Supplementary material for: Unacylated Ghrelin Suppresses Ghrelin-Induced Neuronal Activity in the Hypothalamus and Brainstem of Male Rats
Source: PLoS One. 2014 May 22;9(5):e98180. doi: 10.1371/journal.pone.0098180 (PMC4031147; doi:10.1371/journal.pone.0098180)
Supplement: Table S2 — Analyses of variance data of the various parameters measured in the study. (DOCX) [file pone.0098180.s003.docx]

Table S2: Analyses of variance data of the various parameters measured in the study.

| **Parameter** | **Degrees of freedom between groups** | **Degrees of freedom within groups** | **F value** | **P value** |
| --- | --- | --- | --- | --- |
| Food intake | 3 | 36 | 3.84 | 0.02 |
| Food intake corrected for bw. | 3 | 36 | 4.42 | 0.01 |
| ARC c-Fos IR | 3 | 236 | 13.46 | <0.0001 |
| PVN c-Fos IR | 3 | 243 | 11.33 | 0.0002 |
| NTS c-Fos IR | 3 | 149 | 8.83 | <0.0001 |
| *Mc3r* mRNA | 3 | 17 | 3.41 | 0.034 |
| *Mc4r* mRNA | 3 | 17 | 7.92 | 0.002 |
| *Agrp* mRNA | 3 | 18 | 7.66 | 0.002 |
| *Pomc* mRNA | 3 | 18 | 4.17 | 0.028 |
| *Ucp2* mRNA | 3 | 17 | 2.65 | 0.032 |
| *Bmp8b* mRNA | 3 | 17 | 4.39 | 0.021 |
