# Supplementary material for: Correction: Unacylated Ghrelin Suppresses Ghrelin-Induced Neuronal Activity in the Hypothalamus and Brainstem of Male Rats
Source: PLoS One. 2014 Jul 7;9(7):e102636. doi: 10.1371/journal.pone.0102636 (PMC4085028; doi:10.1371/journal.pone.0102636)
Supplement: File S1 — Originally published, uncorrected article (PDF) [file pone.0102636.s001.pdf]

Unacylated Ghrelin Suppresses Grelin-Induced Neuronal Activity in the Hypothalamus and Brainstem of Male Rats

Darko M. Stevanovic[1,2]*, Aldo Grefhorst[1], Axel P. N. Themmen[1], Vera Popovic[3], Joan Holstege[4], Elize Haasdijk[4], Vladimir Trajkovic[5], Aart-Jan van der Lely[1], Patric J. D. Delhanty[1]*

1 Department of Internal Medicine, Erasmus Medical Center, Rotterdam, The Netherlands, **2** Institute of Medical Physiology, School of Medicine, University of Belgrade, Belgrade, Serbia, **3** Institute of Endocrinology, Diabetes and Diseases of Metabolism, School of Medicine, University of Belgrade, Belgrade, Serbia, **4** Department of Neuroscience, Erasmus Medical Center, Rotterdam, The Netherlands, **5** Institute of Microbiology and Immunology, School of Medicine, University of Belgrade, Belgrade, Serbia

Abstract

Ghrelin, the endogenous growth hormone secretagogue, has an important role in metabolic homeostasis. It exists in two major molecular forms: acylated (AG) and unacylated (UAG). Many studies suggest different roles for these two forms of ghrelin in energy balance regulation. In the present study, we compared the effects of acute intracerebroventricular administration of AG, UAG and their combination (AG+UAG) to young adult Wistar rats on food intake and central melanocortin system modulation. Although UAG did not affect food intake it significantly increased the number of c-Fos positive neurons in the arcuate (ARC), paraventricular (PVN) and solitary tract (NTS) nuclei. In contrast, UAG suppressed AG-induced neuronal activity in PVN and NTS. Central UAG also modulated hypothalamic expression of *Mc4r* and *Bmp8b*, which were increased and *Mc3r*, *Pomc*, *Agrp* and *Ucp2*, which were decreased. Finally, UAG, AG and combination treatments caused activation of c-Fos in POMC expressing neurons in the arcuate, substantiating a physiologic effect of these peptides on the central melanocortin system. Together, these results demonstrate that UAG can act directly to increase neuronal activity in the hypothalamus and is able to counteract AG-induced neuronal activity in the PVN and NTS. UAG also modulates expression of members of the melanocortin signaling system in the hypothalamus. In the absence of an effect on energy intake, these findings indicate that UAG could affect energy homeostasis by modulation of the central melanocortin system.

Citation: Stevanovic DM, Grefhorst A, Themmen APN, Popovic V, Holstege J, et al. (2014) Unacylated Ghrelin Suppresses Grelin-Induced Neuronal Activity in the Hypothalamus and Brainstem of Male Rats. PLoS ONE 9(5): e98180. doi:10.1371/journal.pone.0098180

Editor: Thierry Alquier, CRCHUM-Montreal Diabetes Research Center, Canada

Received February 17, 2014; **Accepted** April 29, 2014; **Published** May 22, 2014

Copyright: © 2014 Stevanovic et al. This is an open-access article distributed under the terms of the Creative Commons Attribution License, which permits unrestricted use, distribution, and reproduction in any medium, provided the original author and source are credited.

Funding: This work was assisted by the ENDO/ESE International Endocrine Scholars Program Fellowship 2011 (DS) supported by the European Society of Endocrinology, and Serbian Ministry of Science and Technological Development proj. no. III 41025. The funders had no role in study design, data collection and analysis, decision to publish, or preparation of the manuscript.

Competing Interests: The authors have declared that no competing interests exist.

* E-mail: dstevano@bidmc.harvard.edu (DS); p.delhanty@erasmusmc.nl (PJDD)

Introduction

The prevalence of obesity and related diseases worldwide has catalyzed the need for a greater understanding of how physiological signals of energy intake and/or energy expenditure converge within the brain to regulate energy homeostasis. The brain melanocortin system represents a fundamental component of centrally regulated energy balance. It consists of circuits of neurons expressing either anorexigenic pro-opiomelanocortin (POMC)-derived melanocortin 3 (MC3) and 4 (MC4) receptor agonists, as well as MC3R and MC4R expressing cells, which are targets of these neurons. The system also includes orexigenic neurons that express the melanocortin receptor inverse agonist agouti-related peptide (AgRP). Distinct populations of AgRP and POMC expressing neurons are found within the arcuate nucleus of the hypothalamus (ARC) and are co-expressed with neuropeptide Y (NPY) and cocaine- and amphetamine-regulated transcript (CART), respectively [1]. These "first order" neurons are able to receive peripheral signals about current energy balance via a wide range of circulating hormones (e.g. leptin, insulin, ghrelin, peptide $YY_{3\text{-}36}$) and nutrients (e.g. glucose, fatty acids, amino acids), mediate anabolic or catabolic effects on energy balance and hence modulate food intake and energy expenditure. Melanocortin neurons in the ARC send projections to downstream "secondary" neuronal populations within proximal nuclei of the hypothalamus, especially to the paraventricular nucleus (PVN). The ARC and PVN, which contain neurons that express MC3R and MC4R, serve as branch points for activation of many central melanocortin-induced circuits involved in body weight regulation [2]. POMC-positive neurons and neural projections are also located within the nucleus of the solitary tract (NTS) of the caudal brainstem. This area receives and integrates both vagal afferent satiation and blood born energy status signals, and issues output commands essential to energy balance control [3–5]. The function of POMC neurons within the NTS may differ significantly from those in the ARC. Only a small number of studies address this issue, but they suggest divergent roles for hindbrain and forebrain POMC neurons in energy homeostasis [6–9].

Ghrelin is a 28-amino acid peptide hormone that can be acylated on its third serine residue (acylated ghrelin, AG) by ghrelin *O*-acyl transferase (GOAT), and is produced predominantly by the gastric oxyntic mucosa in mammals [10–12]. Acylation is required for ghrelin to bind to its receptor, the growth

hormone secretagogue receptor (GHSR) type 1a [13], located in the hypothalamo-pituitary unit, leading to stimulation of food intake and growth hormone (GH) secretion [10]. Recent studies have revealed that central and peripheral administration of AG results in increased NTS activation, suggesting a role for the NTS in mediating the feed-forward mechanisms of food intake [14–16]. However, Kobelt *et al.* (2008) did not find any change in c-Fos positive neurons in the NTS after peripheral administration of AG [17]. Unacylated ghrelin (UAG) also occurs in the circulation [13]. Although UAG does not activate GHSR1a, it has physiological activity [18–24]. A number of studies report that UAG suppresses food intake in rodents both centrally and peripherally [25–27], and the effect is likely mediated via ARC and PVN neurons [26]. At the level of the NTS UAG has been shown to disrupt motor activity in the gastric antrum under fasting conditions, which could potentially modulate food intake [28]. In contrast, Toshinai *et al.* (2006) reported that centrally applied UAG stimulates food intake, while other reports suggest its peripheral administration has no effect on food intake in rodents and humans [18,29,30].

Because it is currently unclear if central UAG has an effect on food intake, we investigated whether central acute administration of AG, UAG or their combination affect neuronal activity in the ARC, PVN and NTS, and hence food intake. Furthermore, to obtain insight into the ability of the ghrelin system to modulate energy expenditure via central mechanisms, we examined changes in hypothalamic mitochondrial uncoupling protein 2 (*Ucp2*) and *Bmp8b* gene expression, as molecules known to regulate thermogenesis and energy balance [31,32].

Materials and Methods

Animals, animal preparation and treatment

The study was performed with 8 week old male Wistar rats (n = 40, body weight = 230±20 g), bred at the Institute of Biomedical Research "Galenika" in Belgrade, Serbia. They were kept in individual metabolic cages under a 12:12 h light-dark cycle, at 22±2°C, and were accustomed to daily handling. Animals received *ad libitum* water and a standard balanced diet (D.D. Veterinarski zavod Subotica, Subotica, Serbia) throughout the experiment.

Animals were anesthetized with intramuscular ketamine (50 mg/kg, Pfizer, New York, NY), xylazine (80 mg/kg, Bayer, Leverkusen, Germany), and surgically equipped with a headset for intracerebroventricular (ICV) injection, consisting of a silastic-sealed 20-gauge cannula positioned in the right lateral cerebral ventricle (1 mm posterior and 1.5 mm lateral to the bregma, and 3 mm below the cortical surface) [33]. A small stainless steel anchor screw was placed at the remote site on the skull. The cannula and screw were cemented to the skull with standard dental acrylic. After surgery, the animals received a single dose of s.c. 0.28 mg/kg buprenorphin (Buprenex; Reckitt Benckiser Healthcare, Mannheim, Germany) followed by a recovery period of one week. Only animals demonstrating progressive weight gain during the recovery period were used in subsequent experiments. Proper ICV cannula placement was verified at 48 hours before conducting any experiment by demonstrating short-latency, heart rate, and drinking responses to a bolus injection of Angiotensin II (50 ng/1 ug). Aspiration of CSF from the guide cannula also was used to indicate correct positioning of the cannula in the lateral ventricle. Animals were randomly divided into 4 groups (control, AG, UAG and AG+UAG groups, n = 10). Animals from the control group were treated ICV with 5 µl of phosphate buffered saline (PBS), while those from the AG and UAG groups received ICV 5 µg of peptide (Neosystem, Strasbourg, France) in 5 µl of PBS. Rats of the combined AG+UAG group were treated ICV with 5 µg of each peptide in a total of 5 µl of PBS. All treatments were administered between 10:00 and 11:00 a.m, and food intake was measured. Differences in food intake were considered statistically significant at $p<0.05$, and considered trends if the p-value was between 0.05 and 0.1. At 2 hrs after ICV injection four animals from each group were deeply anesthetized with isoflurane and transcardially perfused with sterile PBS, followed by 4% of paraformaldehyde. Whole brains were excised, and later used for immunohistochemical studies. At 5 hrs postinjection the remaining six animals in each group were killed by decapitation under deep anesthesia with isoflurane, and hypothalami were collected and stored at −20°C in RNA*later* stabilisation reagent (Qiagen N.V, Venlo, The Netherlands). The samples were transferred to The Netherlands and analyzed at Erasmus MC, Rotterdam. All experimental procedures were approved by the Ethics Committee of the School of Medicine, University of Belgrade, Serbia. All efforts were made to minimize suffering.

Immunohistochemistry

Expression of the proto-oncogene c-Fos was used as a marker for activation of neurons. After perfusion, post-fixation was performed for 1 h in 4% paraformaldehyde (PFA) followed by overnight incubation in 10% sucrose in 0.1 M PBS at 4°C. Subsequently, the dura mater was removed, the tissue was embedded in 10% sucrose in 10% gelatine, and fixed with 10% PFA in 30% sucrose for 2.5 h at room temperature. This was followed by an overnight incubation in 30% sucrose in 0.1 M PBS at 4°C. Serial coronal sections (40 µm) were made using a sliding microtome with a cryostat modification (Leica, Bensheim, Germany). Free-floating sections were processed for immunohistochemistry.

C-Fos immunohistochemistry. Sections were incubated in 10% heat-inactivated normal horse serum (NHS) with 0.5% Triton-X100 in PBS for 1 h, and then incubated for 48 h with a polyclonal rabbit anti-c-Fos antibody (1:15000, Calbiochem, Billerica, MA; PC38) at 4°C, rinsed in PBS (4×10 min) and incubated in 1:200 biotinylated goat anti-rabbit IgG secondary antibody (Sigma-Aldrich, St. Louis, MO, USA) for 1.5 h at room temperature. After washing in PBS (4×10 min), all sections were treated with avidin-biotin complex (ABC Elite Kit, Vector, Burlingame, CA, USA). After washing (6×10 min) the peroxidase component of the ABC complex was visualized using a solution of 0.05% diaminobenzidine tetrachloride and 0.3% H_2O_2. The sections were mounted, air dried overnight, counterstained with thionine for 5 min, dehydrated through a graded series of ethanol and xylene, and coverslipped. Assessment of c-Fos immunoreactive neurons was obtained by counting the number of c-Fos immunopositive nuclei. Neurons with black or dark brown nuclear staining were considered as c-Fos positive. Coronal sections were counted for c-Fos immunopositive staining bilaterally in the ARC (12 sections per rat; bregma -2.12 mm to -3.24 mm), PVN (8 sections per rat; bregma -1.56 mm to -2.08 mm) and NTS (8 sections per rat; bregma -13.68 mm to -14.20 mm), using a Nikon Eclipse E400 photomicroscope. Anatomic correlations were made according to landmarks given in Paxinos and Watson's stereotaxic atlas [34]. The investigator counting the number of c-Fos immunopositive cells was blinded to treatments received by the animals. The average number of c-Fos immunopositive neurons per section for the brain nuclei mentioned above was calculated for four rats per experimental group. C-Fos data are expressed as mean ± SEM and differences between experimental groups were assessed by ANOVA (Tukey's *post hoc* test), with $p<0.05$ considered significant.

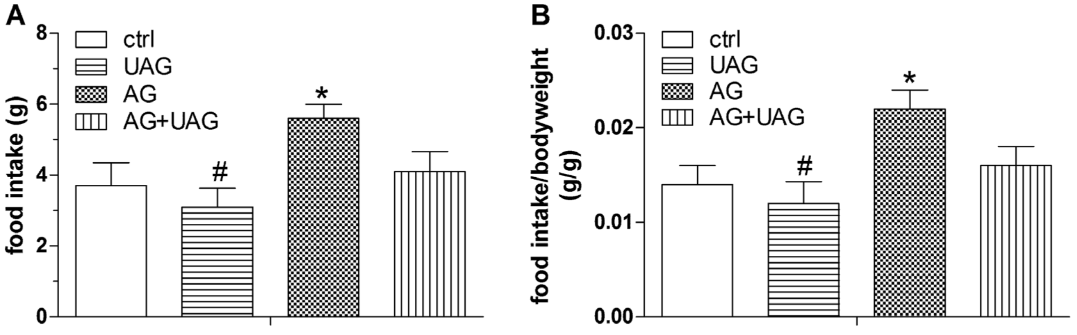

Figure 1. Average food intake (A) and average food intake corrected for body weight (B) 2 hrs after ICV injections of acylated (AG, 5 μg), unacylated (UAG, 5 μg) and combination (AG+UAG, 5 μg of each peptide). Data are mean ± SEM, n = 10 per group, ANOVA, *p<0.05 vs. saline-treated animals, #p<0.05 vs. AG group.
doi:10.1371/journal.pone.0098180.g001

Multi-label immunofluorescence histochemistry and confocal microscopy. Sections were washed (4×15 min) in Tris buffered saline (TBS; 50 mM Tris-Cl, pH 7.5. 150 mM NaCl, pH 7.5), then blocked in TBS, 10% NHS, 0.4% Triton X-100. Sections were then incubated overnight at 4°C in first primary antibody in TBS, 2% NHS, 0.4% Triton X-100 (polyclonal rabbit anti-c-Fos antibody (1:15000; Calbiochem, Billerica, MA, US; PC38)), then washed (4×15 min) in TBS, followed by a 90 min incubation in Cy3-conjugate goat anti-rabbit Fab (1:200; Jackson Immunoresearch Labs Inc., West Grove, PA; 111-167-003). After washing (4×15 min) in TBS, the section were then incubated overnight at 4°C in the second primary antibody in TBS, 2% NHS, 0.4% Triton X-100 (anti-POMC antibody (1:5000; Phoenix Pharmaceuticals Inc. Burlingame, CA; H-029-30)), then after washing in TBS, incubated 90 min in Alexa Fluor 488-conjugated donkey anti-rabbit antibody (1:200; Jackson Immunoresearch Labs Inc.; 711-545-152). Finally, sections were washed 1×10 min in TBS, 1×10 min in PBS, then stained with DAPI. Sections were then mounted in Vectashield on glass slides. Images were acquired with a Zeiss LSM 700 laser-scanning confocal microscope and a 40× oil-immersion objective lens. Levels of gain and laser power were selected to allow optimal visualization of the fluorophores. Each image was saved at a resolution of 1024×1024 pixels.

Quantitative PCR

Six separate hypothalamic samples were used for RNA isolation. Quantitative PCR was performed using a qPCR Core kit for SYBR Green I (Eurogentec, The Netherlands). Gene specific primers were designed to span introns. The sequences forward and reverse were as follows: β-actin, 5′-CCCTGGCT-CCTAGCACCAT and 5′-GAGCCACCAATCCACACAGA, Hprt, 5′-TGGTCAAGCAGTACAGCCCCA and 5′- GGCCT-GTATCCAACACTTCGAGAGG; Mc3r, 5′-GCAACCGGAG-TGGCAGTGGG and 5′-GGGGAGTGCAGGTTGCCGTT; Mc4r, 5′-CTCCCGGGCACGGGTACCAT and 5′-AACGG-GGCCCAGCAGACAAC; Agrp, 5′-AGACAGCAGCAGACC-GAGCAGA and 5′-CACAGCGACGCGGAGAACGA; Pomc, 5′-AGACGTGTGGAGCTGGTGCC and 5′-CTGCAGGCC-CGGATGCAAGC; Ucp2, 5′-ATGAGCTTTGCCTCCGTCC-GC and 5′-GGGCACCTGTGGTGCTACCTG; Bmp8b, 5′-CCACGCCACTATGCAGGCCC and 5′-GGCACTCAGCTT-GGTGGGCA. Gene expression was calculated using the ΔC_t method relative to the mean of 2 housekeeping genes (*Actb* and *Hprt*), and mean values +/- SEM are shown in Table S1.

Statistical analyses

All data were analyzed by ANOVA using Tukey's *post hoc* test, with effects being considered significant at $p<0.05$. Degrees of freedom, F-values and p-values of the analyses are summarized in Table S2.

Results

Food intake

To evaluate the immediate effect of central AG, UAG or their combination treatment, we assessed food intake in all groups (n = 10 per group) 2 hrs after ICV injection (Fig. 1). In comparison to average food intake in the control group (3.7 g±0.65), AG caused a significant increase in average food intake (5.6 g±0.40), as expected, while there was no significant change in food intake in the UAG (3.1 g±0.53) group compared to controls. Also, a significant difference in food intake was observed between the AG and UAG treated groups, while UAG noticeably reduced the appetitive response to AG when given in combination, although this effect only showed a trend (Fig. 1; AG+UAG treatment, 4.1 g±0.56, p = 0.08).

C-Fos immunoreactivity in the ARC, PVN and NTS

To gain additional insight into the possible interaction between AG and UAG in regulating central neuronal pathways involved in energy homeostasis, we examined whether AG, UAG or combined treatment induce changes in c-Fos immunoreactivity in hypothalamic ARC and PVN as well as in the NTS of the brainstem (see representative micrographs in Figs. 2, 3 and 4A–D). Central AG, UAG and combined treatments all significantly induced c-Fos immunoactivity in all examined brain regions (Figs. 2, 3 and 4, see histograms for quantitative data). Although AG caused a significantly greater induction of c-Fos immunoreactivity than UAG, the effects of AG and the combined treatments were not significantly different.

To investigate the biological relevance of the regulation of this neuronal activation by ghrelin peptides in relation to melanocortin signaling, we used multi-label immunofluorescence to discover if the peptides, and UAG treatment in particular, caused c-Fos immunoreactivity in POMC immunopositive neurons. We observed that POMC neurons in the arcuate show co-expression of c-Fos following treatment with these peptides (Fig. 5). We then assessed the relative expression of c-Fos in POMC positive cells, denoting activation of these cells by the different treatments (figure S1A), as well as the relative expression of POMC in c-Fos positive cells (figure S1B). We found that UAG treatment caused a trend to

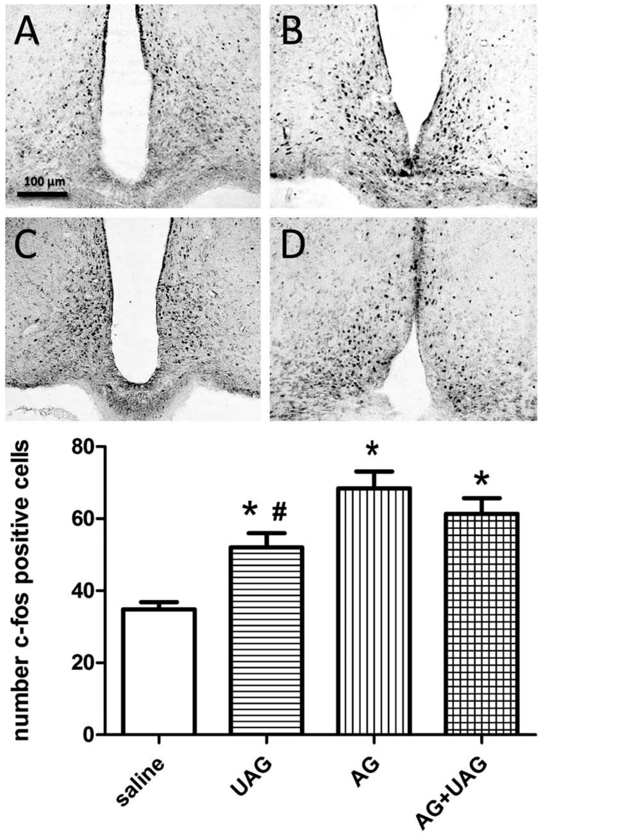

Figure 2. C-Fos immunoreactivity in the arcuate nucleus (ARC) two hrs after ICV injections of vehicle (A) UAG (B), AG (C) or AG+UAG (D). The scale bar applies to all images which are representative of sections from 4 rats. C-Fos positive nuclei were counted in 12 sections from 4 rats and these quantitative data are presented in the histogram (*, p<0.01 v. saline; #, p<0.05 v. AG). Color images were corrected for color balance and contrast before conversion to grayscale.
doi:10.1371/journal.pone.0098180.g002

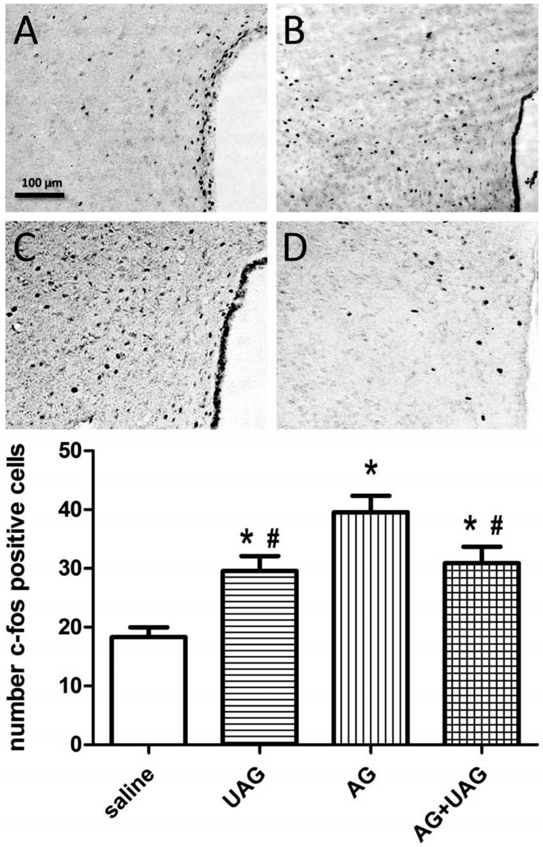

Figure 3. C-Fos immunoreactivity in the paraventricular nucleus (PVN) two hrs after ICV injections of vehicle (A) UAG (B), AG (C) or AG+UAG (D). The scale bar applies to all images. C-Fos positive nuclei were counted in 8 sections from 4 rats and these quantitative data are presented in the histogram (*, p<0.01 v. saline; #, p<0.05 v. AG). Color images were corrected for color balance and contrast before conversion to grayscale.
doi:10.1371/journal.pone.0098180.g003

induce c-Fos immunorectivity in POMC immunoreactive cell bodies, and that this was significantly greater than the levels of c-Fos in AG+UAG treated animals. The distribution of POMC positive c-Fos expressing cells was not affected by treatment.

Hypothalamic *Mc4r*, *Mc3r*, *Agrp*, *Pomc*, *Ucp2* and *Bmp8b* gene expression

To determine a possible role for UAG in modulating the effect of AG on the hypothalamic melanocortin system, we examined hypothalamic *Mc4r*, *Mc3r*, *Agrp* and *Pomc* gene expression following ICV injection of the peptides. *Mc4r* mRNA was significantly ($p<0.05$) increased in the UAG and AG+UAG treated groups, while there was no change in *Mc4r* mRNA expression in the group treated with AG alone (Fig. 6A–D). In contrast, *Mc3r* mRNA expression was significantly decreased by UAG treatment when compared to controls ($p<0.05$) and AG treated animals ($p = 0.04$). Both *Agrp* and *Pomc* gene expression were significantly decreased in UAG and AG+UAG groups (*Agrp*-UAG, $p = 0.03$; *Agrp*-AG+UAG = $p<0.03$; *Pomc*-UAG $p = 0.0004$; *Pomc*-AG+UAG $p = 0.004$). Central AG treatment did not affect expression of these two important components of the melanocortin system. Gene expression of other important players in the central melanocortin system, *Npy* and *Cart*, were not significantly altered by AG and/or UAG treatment (data not shown).

To examine further a possible role for both forms of ghrelin in energy expenditure, we examined changes in hypothalamic *Ucp2* and *Bmp8b* gene expression 5 hrs post-treatment (Fig. 7A and B). Results showed a significant decrease in hypothalamic *Ucp2* mRNA in both UAG and AG+UAG groups compared to control animals ($p<0.01$) and AG groups alone. UAG treatment alone significantly increased *Bmp8b* mRNA expression ($p<0.05$) in comparison to control and AG+UAG groups.

Discussion

A large body of evidence shows that AG's most impressive impact on mammalian energy balance appears to be an almost instant induction of food intake when administered in pharmacological doses, even in satiated animals [35,36]. On the other hand, currently available data regarding UAG effects on food intake are inconsistent [37]. A significant anorexigenic effect of UAG was found in fasted and animals fed *ad libitum* during the dark phase and in food-restricted rats throughout the light phase has been described [38]. However, Toshinai and co-workers (2006) did not observe an anorexigenic effect of UAG during the light phase [29]. They also found no significant reduction in food intake in fasted and *ad libitum* fed animals after peripheral UAG administration [30,38]. These differences in UAG's effect on food intake could be due to variability in experimental setup, time of measurement

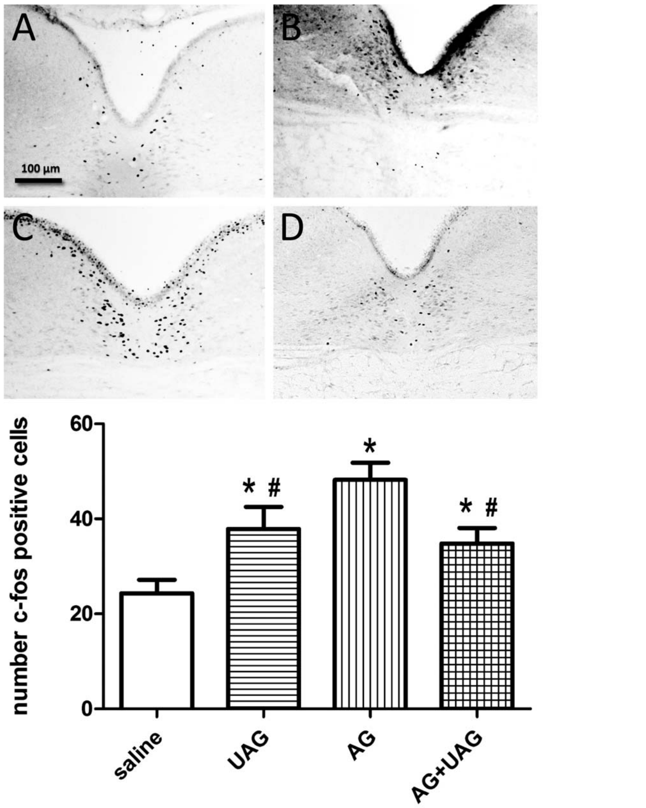

Figure 4. C-Fos immunoreactivity in the nucleus of the solitary tract (NTS) two hrs after ICV injections of vehicle (A) UAG (B), AG (C) or AG+UAG (D). The scale bar applies to all images. C-Fos positive nuclei were counted in 8 sections from 4 rats and these quantitative data are presented in the histogram (*, $p<0.01$ v. saline; #, $p<0.05$ v. AG). Color images were corrected for color balance and contrast before conversion to grayscale.
doi:10.1371/journal.pone.0098180.g004

(light vs. dark phase) and/or circadian rhythmicity. We found that UAG showed a trend to inhibit AG mediated induction of food intake. This is comparable to the results of Inhoff *et al.* (2008) who found that AG-induced food intake was diminished by i.p. injection of UAG [27]. The inhibitory effect of i.p. UAG on ICV AG-induced increase in food intake was observed at a dose that had no effect on food intake in freely fed rats monitored during the light phase, 5 h after treatment [27]. Since our measurements were performed on rats that had been fed *ad libitum* during the light phase and were likely satiated, it should be noted that it is rather difficult to detect an anorexigenic effect of a satiety hormone under these conditions. This could explain why we failed to observe a significant satiating effect of UAG.

An inhibitory effect of UAG on AG has also been shown in goldfish (*Carrassius auratus*), where UAG administered either ICV or i.p. substantially reduced AG-induced food intake, while having no effect if its own [39].

To gain additional insight into the role of AG-UAG system in central melanocortin neuronal pathways involved in energy homeostasis, we examined whether AG, UAG or their combination treatment induced changes in c-Fos immunoreactivity in the ARC and PVN as well as in the NTS of the brainstem. Our results show that central AG, UAG and combined treatments rapidly induced neuronal activity in all examined brain regions, and UAG reduced AG-induced neuronal activity in the PVN and NTS. Furthermore, we performed c-Fos and POMC double-labeling

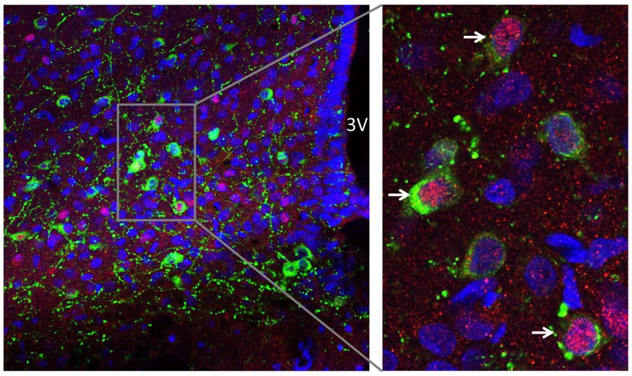

Figure 5. POMC and c-Fos are co-expressed in neurons of the arcuate nucleus of the hypothalamus following UAG treatment. POMC and c-Fos immunoreactivity was identified in sections of the hypothalamus using multi-label immunofluorescence immunohistochemistry. Separate nuclear (DAPI, blue), POMC (green), c-Fos (red) and composite (merge) confocal laser-scanning microscope images are shown from a representative section. The arrowhead indicates a neuron that contains both POMC and c-Fos immunoreactivity. The scale bar represents 20 µm.
doi:10.1371/journal.pone.0098180.g005

immunofluorescence to show that UAG, AG and combination treatments caused the appearance of c-Fos immunoreactivity in POMC-positive neurons. Intriguingly, UAG, but not AG, appeared to induce c-Fos in POMC cell bodies, and this effect of UAG was blocked by combined AG treatment. This fits with activation of anorexigenic circuits in the arcuate by UAG and demonstrate a possible interaction with AG. However, this possible mechanism of action by UAG, and interaction with AG, needs to be verified using POMC-EGFP knock-in mice [40].

Two other studies in rodents also describe increased neuronal activity (c-Fos positive neurons) in the ARC and in the PVN following ICV UAG treatment, while the same treatment had no effect on neuronal activity in the NTS [25,38]. According to these latter results, UAG is involved in the regulation of the synthesis of anorexigenic CART and urocortin 1 in the hypothalamus. However, we did not observe any changes in hypothalamic *Cart* (or *Npy*) gene expression (data not shown) after central AG, UAG and their combined treatment, while hypothalamic *Pomc* and *Agrp* gene expression was suppressed in both UAG and AG+UAG, but not AG, when compared to the control group. Down-regulation of *Pomc* mRNA may indicate the down-regulation of a potent suppressor of food intake (α-melanocyte-stimulating hormone, α-MSH), but also β-endorphin which, on the other hand, stimulates feeding [41]. Thus, an alternative interpretation may be that the down-regulation of *Pomc* mRNA, with subsequent decreased levels of β-endorphin, results in less rewarding signals through the opioid pathway by UAG. Importantly, AG by itself had no effect on these genes, which were only regulated by UAG alone or when in combination with AG, suggesting a specific effect of UAG.

Ucp2 has been shown to be an important negative regulator of reactive oxygen species in the hypothalamus [31]. Interestingly, AgRP (orexigenic) derived action potentials are suppressed by raised ROS, whereas POMC-related (anorexigenic) signals are induced [42]. UAG suppressed *Ucp2* gene expression, therefore we speculate that UAG causes increased levels of ROS in hypothalamic neurons, and if so this would stimulate anorexigenic pathways, in opposition to the effects of AG. Further work is required to confirm this possibility.

To further explore the role of UAG and its interaction with AG in the regulation of the central melanocortin system, we

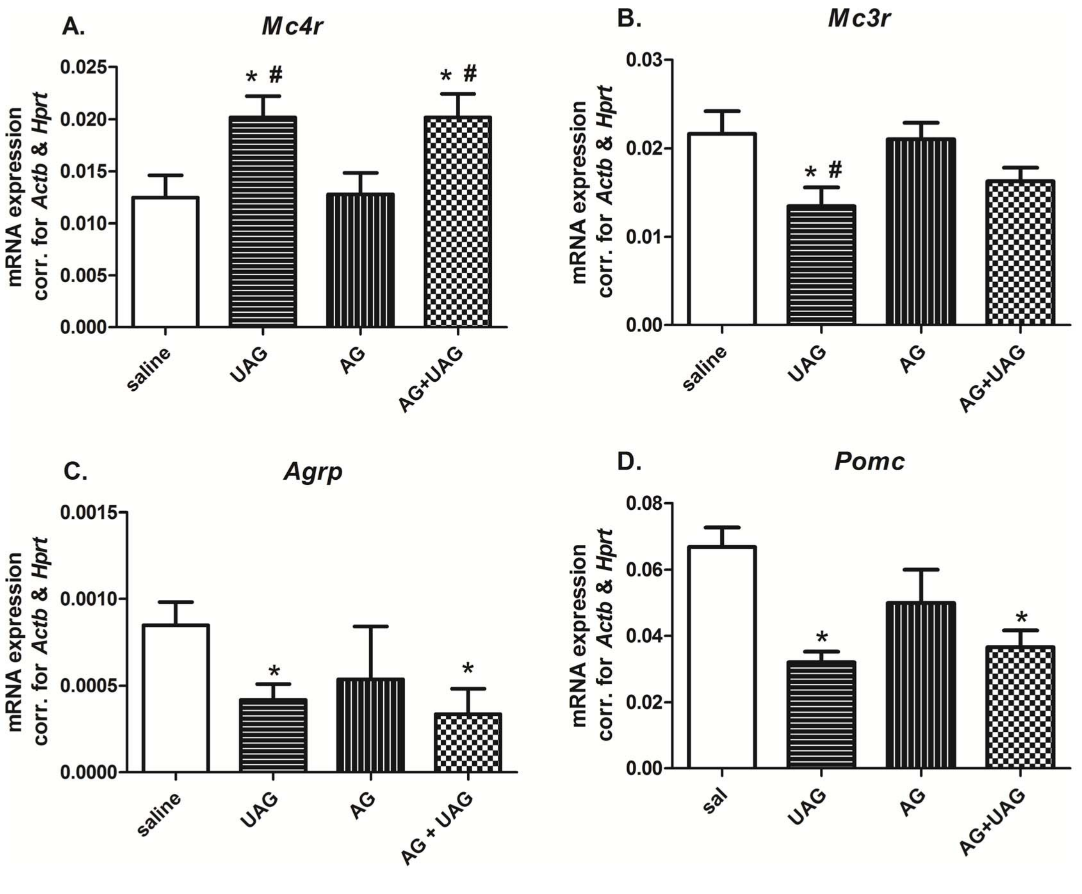

Figure 6. The effects of central AG, UAG and combination of AG and UAG treatment on hypothalamic *Mc4r* (A), *Mc3r* (B), *Agrp* (C) and *Pomc* (D) gene expression, corrected for *Hprt* and *Actb*. Data are mean ± SEM, n = 6 per group, ANOVA, $^{*}p<0.05$ vs. saline-treated animals, $^{\#}p<0.05$ vs. AG group.
doi:10.1371/journal.pone.0098180.g006

investigated whether AG, UAG and their combination affect hypothalamic *Mc4r* and *Mc3r* gene expression. Although hypothalamic *Pomc* gene expression was decreased, this is likely compensated for by a significant increase in *Mc4r* after UAG treatment. By increasing MC4R expression, UAG may in effect amplify the POMC driven neuronal signal to induce energy expenditure. Also, possible effects of UAG on complex circuits (e.g. the melanocortin system) regulating energy expenditure in the brain and those regulating appetite may involve other central and peripheral factors/systems, which could separately act on these circuits to modulate overall energy homeostasis. In agreement with this hypothesis, we have recently shown that peripheral UAG infusion can induced expression of genes, such as *Ucp1*, *Pgc1a* and *Bmp8b* in brown adipose tissue of mice on a high fat diet [18].

MC4R is directly activated by α-MSH from POMC neurons, an effect that is inhibited by AgRP from orexigenic neurons in the ARC. MC4R-deficient mice are hyperphagic and obese [43,44]. It has also been shown that stimulation of AgRP-producing neurons involves a melanocortin receptor-independent mechanism to increase food intake, whereas POMC stimulation requires intact melanocortin receptor to reduce food intake [45]. Feeding stimulatory effects of AgRP is instead likely to occur via GABA release/GABA-ergic signaling [46,47]. Taken together, these data point to a distributed control of MC4R signaling within the hypothalamus and between forebrain and hindbrain in regulating energy balance and food intake.

It is known that the melanocortin system modulates energy expenditure via the sympathetic nervous system (SNS). Recently, it was shown that BMP8B has an important role in energy expenditure, by acting both centrally in the hypothalamus and peripherally in brown adipose tissue (BAT) [32]. *Bmp8b* gene expression has been described in the arcuate and ventromedial regions of the hypothalamus [32]. Both regions are important in regulation of BAT activity and energy expenditure. Using *Bmp8b* deficient mice, it was shown that *Bmp8b* is required for the response of BAT to adrenergic stimulation by acting at the hypothalamic level to increase sympathetic output. In this study we observed that hypothalamic *Bmp8b* gene expression is significantly increased by UAG central administration alone. Together with the increase in hypothalamic *Mc4r* gene expression, we could speculate that UAG could affect energy expenditure by upregulating *Mc4r* and *Bmp8b* gene expression in the hypothalamus. Interestingly, however, combined AG+UAG treatment had no effect on *Bmp8b* expression. One possibility is that AG, when it is co-infused, is able to prevent UAG-induced *Bmp8b* mRNA expression in the brain, but by itself has no effect. Like MC4R, MC3R is expressed throughout the brain, especially in the ARC and NTS [48], and endogenous melanocortins activate this receptor [49], suggesting potentially redundant or overlapping actions of both receptors. However, a number of studies suggest that selective MC3R agonists actually stimulate food intake and, unlike MC4R-deleted mice, MC3R-deleted mice are hypophagic [50]. MC3R, but not MC4R, mRNA is expressed in half of the

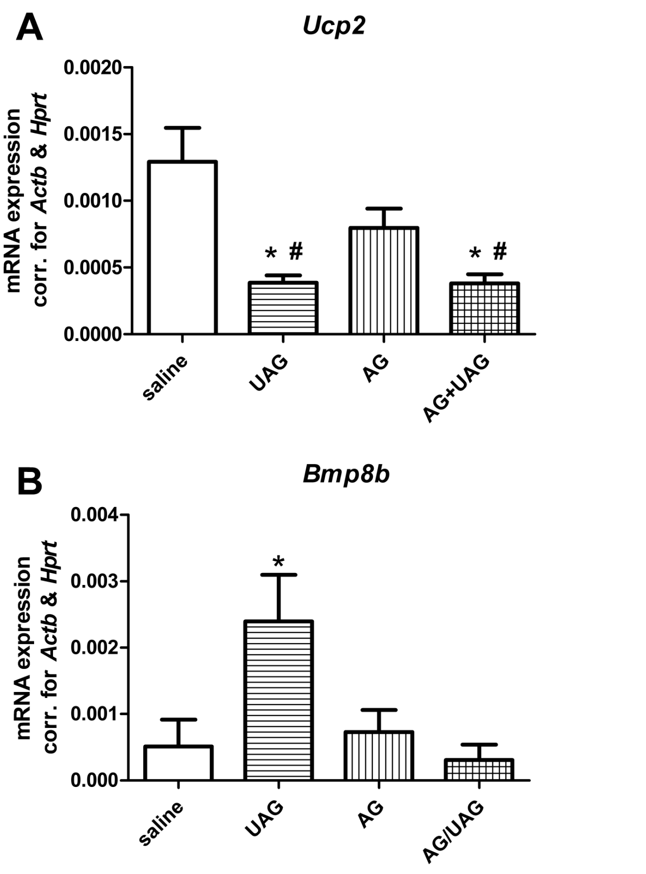

Figure 7. The effects of central AG, UAG and combination of AG and UAG treatment on hypothalamic *Ucp2* (A) and *Bmp8B* (B) gene expression, corrected for *Hprt* and *Actb*. Data are mean ± SEM, n = 6 per group, ANOVA, $^{*}p<0.05$ vs. saline-treated animals, $^{\#}p<0.05$ vs. AG group.
doi:10.1371/journal.pone.0098180.g007

POMC and AgRP neurons in the ARC [2]. The role of MC3R in these neurons is thought to be auto-inhibitory. They serve as messengers within the ARC and between the ARC and PVN to maintain melanocortin tone, and regulate AgRP/POMC activity via inhibitory GABA-ergic terminals to suppress NPY/AgRP signaling and/or direct activation of POMC signaling [40].

In conclusion, our data indicate a new role for the central ghrelin system in energy balance, in which the unacylated form of ghrelin increases neuronal activity directly and independently of AG. We observed this effect not only in the hypothalamus but also in the brainstem, increasing neuronal activity in the NTS. We also show that UAG can suppress AG-induced neuronal activation in the PVN and NTS, and blunt AG-induced food intake. UAG affected hypothalamic gene expression of several main players in the energy balance regulation, decreasing *Pomc*, *Agrp*, *Mc3r* and *Ucp2*, while increasing *Mc4r* and *Bmp8b* gene expression levels, suggesting a role in induction of energy expenditure via the central melanocortin system. This study also shows that UAG does have biological activity, although its mechanism of action remains to be determined at the cellular level. Further insight is needed to understand the etiology and pathogenesis of human diseases characterized by disturbances of energy balance, such as obesity. Our study further highlights the interplay between AG, UAG and the central melanocortin-ghrelin system in controlling energy homeostasis and, ultimately, body weight.

Supporting Information

Figure S1 A. Co-localization of POMC/c-Fos after saline (A), AG (B), UAG (C) and AG+UAG (D) acute central treatment. POMC (green) and c-Fos (red) immunoreactivity was identified in sections of the hypothalamus using multi-label immunofluorescence immunohistochemistry. Nuclear staining (DAPI) is blue. Composite confocal laser-scanning microscope images are shown from a representative sections. The scale bar represents 20 μm. E. POMC/c-Fos co-localization ratio after saline, AG, UAG and combine treatment. Data are presented and mean ± SEM, * $p<0.05$ vs. saline.
(TIF)

Table S1 Cycle threshold (Ct) values for the mRNA species amplified.
(DOCX)

Table S2 Analyses of variance data of the various parameters measured in the study.
(DOCX)

Acknowledgments

The authors wish to thank Martin Huisman from the Department of Internal Medicine, Erasmus MC for excellent technical assistance.

Author Contributions

Conceived and designed the experiments: DS APNT VT AJL PJDD. Performed the experiments: DS. Analyzed the data: DS AG APNT VP JH EH VT AJL PJDD. Contributed reagents/materials/analysis tools: AG APNT JH EH VT AJL. Wrote the paper: DS. Revised manuscript: PJDD.

References

1. Cone RD (2005) Anatomy and regulation of the central melanocortin system. Nat Neurosci 8: 571–578.
2. De Jonghe BC, Hayes MR, Bence KK (2011) Melanocortin control of energy balance: evidence from rodent models. Cell Mol Life Sci 68: 2569–2588.
3. Hayes MR, Skibicka KP, Leichner TM, Guarnieri DJ, DiLeone RJ, et al. (2010) Endogenous leptin signaling in the caudal nucleus tractus solitarius and area postrema is required for energy balance regulation. Cell Metab 11: 77–83.
4. Skibicka KP, Grill HJ (2009) Hindbrain leptin stimulation induces anorexia and hyperthermia mediated by hindbrain melanocortin receptors. Endocrinology 150: 1705–1711.
5. Skibicka KP, Grill HJ (2009) Hypothalamic and hindbrain melanocortin receptors contribute to the feeding, thermogenic, and cardiovascular action of melanocortins. Endocrinology 150: 5351–5361.
6. Li G, Zhang Y, Rodrigues E, Zheng D, Matheny M, et al. (2007) Melanocortin activation of nucleus of the solitary tract avoids anorectic tachyphylaxis and induces prolonged weight loss. Am J Physiol Endocrinol Metab 293: E252–258.
7. Ellacott KL, Halatchev IG, Cone RD (2006) Characterization of leptin-responsive neurons in the caudal brainstem. Endocrinology 147: 3190–3195.
8. Huo L, Grill HJ, Bjorbaek C (2006) Divergent regulation of proopiomelanocortin neurons by leptin in the nucleus of the solitary tract and in the arcuate hypothalamic nucleus. Diabetes 55: 567–573.
9. Zhang Y, Rodrigues E, Gao YX, King M, Cheng KY, et al. (2010) Pro-opiomelanocortin gene transfer to the nucleus of the solitary track but not arcuate nucleus ameliorates chronic diet-induced obesity. Neuroscience 169: 1662–1671.
10. Kojima M, Hosoda H, Date Y, Nakazato M, Matsuo H, et al. (1999) Ghrelin is a growth-hormone-releasing acylated peptide from stomach. Nature 402: 656–660.
11. Gutierrez JA, Solenberg PJ, Perkins DR, Willency JA, Knierman MD, et al. (2008) Ghrelin octanoylation mediated by an orphan lipid transferase. Proc Natl Acad Sci U S A 105: 6320–6325.
12. Yang J, Brown MS, Liang G, Grishin NV, Goldstein JL (2008) Identification of the Acyltransferase that Octanoylates Ghrelin, an Appetite-Stimulating Peptide Hormone. Cell 132: 387–396.

13. Delhanty PJ, Neggers SJ, van der Lely AJ (2012) Mechanisms in endocrinology: Ghrelin: the differences between acyl- and des-acyl ghrelin. Eur J Endocrinol 167: 601–608.
14. Faulconbridge LF, Grill HJ, Kaplan JM, Daniels D (2008) Caudal brainstem delivery of ghrelin induces fos expression in the nucleus of the solitary tract, but not in the arcuate or paraventricular nuclei of the hypothalamus. Brain Res 1218: 151–157.
15. Lawrence CB, Snape AC, Baudoin FM, Luckman SM (2002) Acute central ghrelin and GH secretagogues induce feeding and activate brain appetite centers. Endocrinology 143: 155–162.
16. Takayama K, Johno Y, Hayashi K, Yakabi K, Tanaka T, et al. (2007) Expression of c-Fos protein in the brain after intravenous injection of ghrelin in rats. Neurosci Lett 417: 292–296.
17. Kobelt P, Wisser AS, Stengel A, Goebel M, Inhoff T, et al. (2008) Peripheral injection of ghrelin induces Fos expression in the dorsomedial hypothalamic nucleus in rats. Brain Res 1204: 77–86.
18. Delhanty PJ, Huisman M, Baldeon-Rojas LY, van den Berge I, Grefhorst A, et al. (2013) Des-acyl ghrelin analogs prevent high-fat-diet-induced dysregulation of glucose homeostasis. FASEB J 27: 1690–1700.
19. Heijboer AC, Pijl H, Van den Hoek AM, Havekes LM, Romijn JA, et al. (2006) Gut-brain axis: regulation of glucose metabolism. J Neuroendocrinol 18: 883–894.
20. Muccioli G, Baragli A, Granata R, Papotti M, Ghigo E (2007) Heterogeneity of ghrelin/growth hormone secretagogue receptors. Toward the understanding of the molecular identity of novel ghrelin/GHS receptors. Neuroendocrinology 86: 147–164.
21. Favaro E, Granata R, Miceli I, Baragli A, Settanni F, et al. (2012) The ghrelin gene products and exendin-4 promote survival of human pancreatic islet endothelial cells in hyperglycaemic conditions, through phosphoinositide 3-kinase/Akt, extracellular signal-related kinase (ERK)1/2 and cAMP/protein kinase A (PKA) signalling pathways. Diabetologia 55: 1058–1070.
22. Granata R, Settanni F, Julien M, Nano R, Togliatto G, et al. (2012) Des-acyl ghrelin fragments and analogues promote survival of pancreatic beta-cells and human pancreatic islets and prevent diabetes in streptozotocin-treated rats. J Med Chem 55: 2585–2596.
23. Togliatto G, Trombetta A, Dentelli P, Baragli A, Rosso A, et al. (2010) Unacylated Ghrelin Rescues Endothelial Progenitor Cell Function in Individuals with Type 2 Diabetes. Diabetes 59: 1016–1025.
24. Delhanty PJ, Sun Y, Visser JA, van Kerkwijk A, Huisman M, et al. (2010) Unacylated ghrelin rapidly modulates lipogenic and insulin signaling pathway gene expression in metabolically active tissues of GHSR deleted mice. PLoS One 5: e11749.
25. Asakawa A, Inui A, Fujimiya M, Sakamaki R, Shinfuku N, et al. (2005) Stomach regulates energy balance via acylated ghrelin and desacyl ghrelin. Gut 54: 18–24.
26. Chen CY, Chao Y, Chang FY, Chien EJ, Lee SD, et al. (2005) Intracisternal des-acyl ghrelin inhibits food intake and non-nutrient gastric emptying in conscious rats. Int J Mol Med 16: 695–699.
27. Inhoff T, Monnikes H, Noetzel S, Stengel A, Goebel M, et al. (2008) Desacyl ghrelin inhibits the orexigenic effect of peripherally injected ghrelin in rats. Peptides 29: 2159–2168.
28. Fujimiya M, Asakawa A, Ataka K, Kato I, Inui A (2008) Different effects of ghrelin, des-acyl ghrelin and obestatin on gastroduodenal motility in conscious rats. World J Gastroenterol 14: 6318–6326.
29. Toshinai K, Yamaguchi H, Sun Y, Smith RG, Yamanaka A, et al. (2006) Des-acyl Ghrelin Induces Food Intake by a Mechanism Independent of the Growth Hormone Secretagogue Receptor. Endocrinology 147: 2306–2314.
30. Neary NM, Druce MR, Small CJ, Bloom SR (2006) Acylated ghrelin stimulates food intake in the fed and fasted states but desacylated ghrelin has no effect. Gut 55: 135.
31. Diano S, Horvath TL (2012) Mitochondrial uncoupling protein 2 (UCP2) in glucose and lipid metabolism. Trends Mol Med 18: 52–58.
32. Whittle AJ, Carobbio S, Martins L, Slawik M, Hondares E, et al. (2012) BMP8B Increases Brown Adipose Tissue Thermogenesis through Both Central and Peripheral Actions. Cell 149: 871–885.
33. Starcevic VP, Morrow BA, Farner LA, Keil LC, Severs WB (1988) Long-term recording of cerebrospinal fluid pressure in freely behaving rats. Brain Res 462: 112–117.
34. Paxinos G, Watson C (2004) The rat brain in stereotaxic coordinates. San Diego: Academic Press.
35. Tschöp M, Smiley DL, Heiman ML (2000) Ghrelin induces adiposity in rodents. Nature 407: 908–913.
36. Kirchner H, Heppner KM, Tschop MH (2012) The role of ghrelin in the control of energy balance. Handb Exp Pharmacol: 161–184.
37. Inhoff T, Wiedenmann B, Klapp BF, Monnikes H, Kobelt P (2009) Is desacyl ghrelin a modulator of food intake? Peptides 30: 991–994.
38. Chen CY, Inui A, Asakawa A, Fujino K, Kato I, et al. (2005) Des-acyl ghrelin acts by CRF type 2 receptors to disrupt fasted stomach motility in conscious rats. Gastroenterology 129: 8–25.
39. Matsuda K, Miura T, Kaiya H, Maruyama K, Shimakura S, et al. (2006) Regulation of food intake by acyl and des-acyl ghrelins in the goldfish. Peptides 27: 2321–2325.
40. Cowley MA, Smart JL, Rubinstein M, Cerdan MG, Diano S, et al. (2001) Leptin activates anorexigenic POMC neurons through a neural network in the arcuate nucleus. Nature 411: 480–484.
41. Silva RM, Hadjimarkou MM, Rossi GC, Pasternak GW, Bodnar RJ (2001) Beta-endorphin-induced feeding: pharmacological characterization using selective opioid antagonists and antisense probes in rats. J Pharmacol Exp Ther 297: 590–596.
42. Andrews ZB, Liu ZW, Walllingford N, Erion DM, Borok E, et al. (2008) UCP2 mediates ghrelin's action on NPY/AgRP neurons by lowering free radicals. Nature 454: 846–851.
43. Huszar D, Lynch CA, Fairchild-Huntress V, Dunmore JH, Fang Q, et al. (1997) Targeted disruption of the melanocortin-4 receptor results in obesity in mice. Cell 88: 131–141.
44. Pritchard LE, White A (2007) Neuropeptide processing and its impact on melanocortin pathways. Endocrinology 148: 4201–4207.
45. Aponte Y, Atasoy D, Sternson SM (2011) AGRP neurons are sufficient to orchestrate feeding behavior rapidly and without training. Nat Neurosci 14: 351–355.
46. Wu Q, Boyle MP, Palmiter RD (2009) Loss of GABAergic signaling by AgRP neurons to the parabrachial nucleus leads to starvation. Cell 137: 1225–1234.
47. Wu Q, Palmiter RD (2011) GABAergic signaling by AgRP neurons prevents anorexia via a melanocortin-independent mechanism. Eur J Pharmacol 660: 21–27.
48. Roselli-Rehfuss L, Mountjoy KG, Robbins LS, Mortrud MT, Low MJ, et al. (1993) Identification of a receptor for gamma melanotropin and other proopiomelanocortin peptides in the hypothalamus and limbic system. Proc Natl Acad Sci U S A 90: 8856–8860.
49. Fong TM, Mao C, MacNeil T, Kalyani R, Smith T, et al. (1997) ART (protein product of agouti-related transcript) as an antagonist of MC-3 and MC-4 receptors. Biochem Biophys Res Commun 237: 629–631.
50. Chen AS, Marsh DJ, Trumbauer ME, Frazier EG, Guan XM, et al. (2000) Inactivation of the mouse melanocortin-3 receptor results in increased fat mass and reduced lean body mass. Nat Genet 26: 97–102.
